# Supplementary figures and images for: Hypoxia triggers collective aerotactic migration in Dictyostelium discoideum
Source: eLife. 2021 Aug 20;10:e64731. doi: 10.7554/eLife.64731 (PMC8378850; doi:10.7554/eLife.64731)

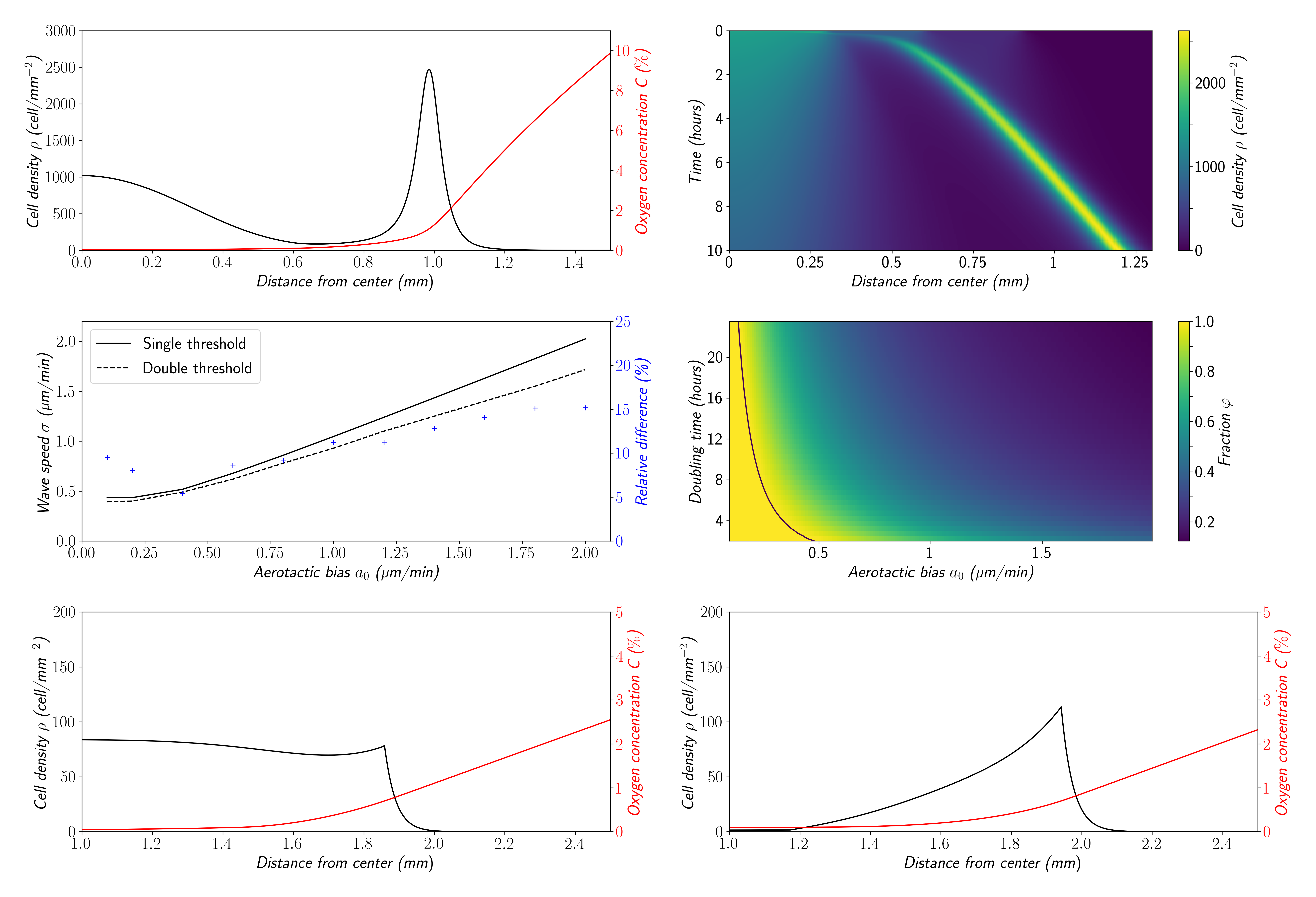

Supplement: Figure 6—source data 1. [file elife-64731-fig6-data1.zip › Fig6/Fig6.png]

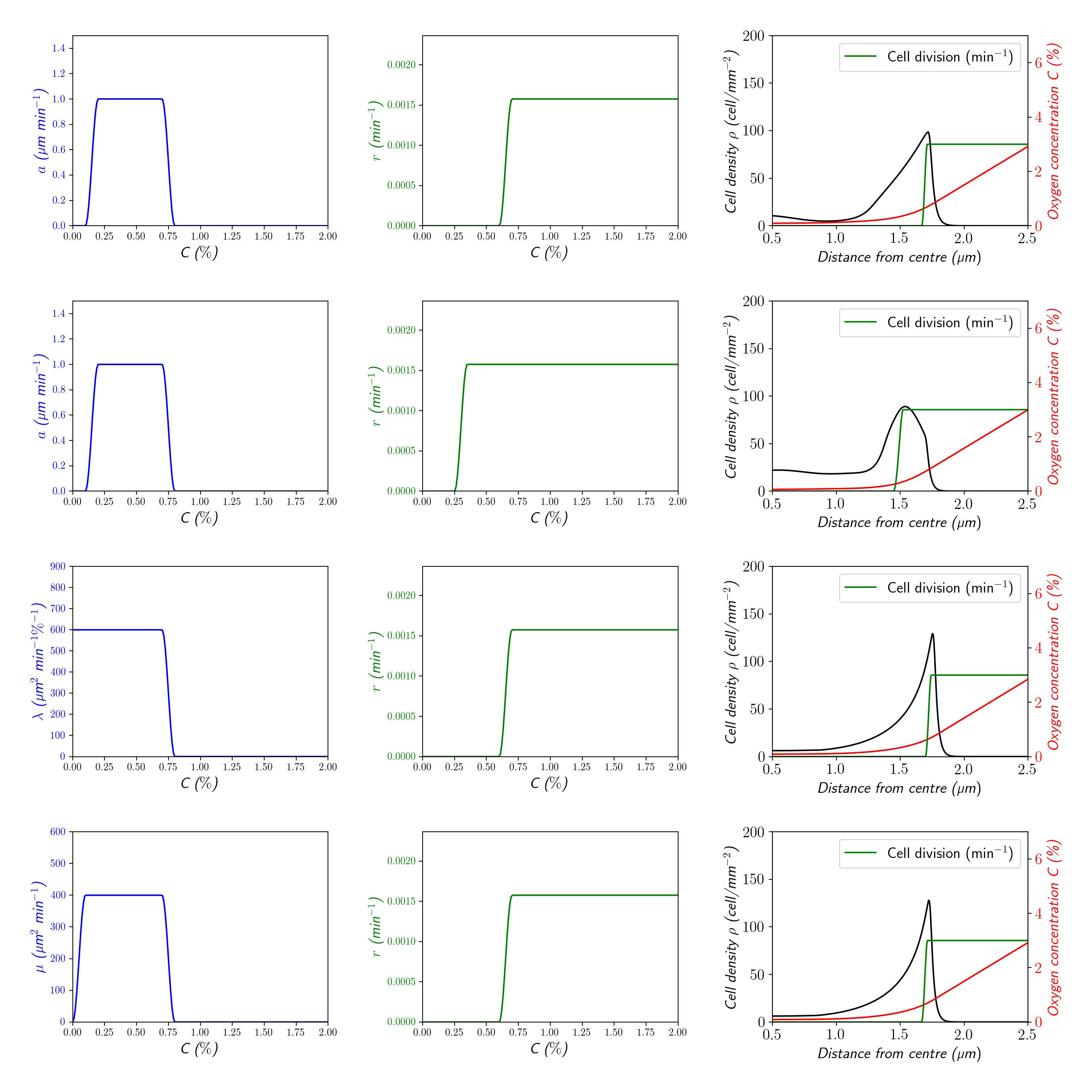

Supplement: Figure 6—figure supplement 1—source data 1. [file elife-64731-fig6-figsupp1-data1.zip › FigSI17/FigSI17.png]

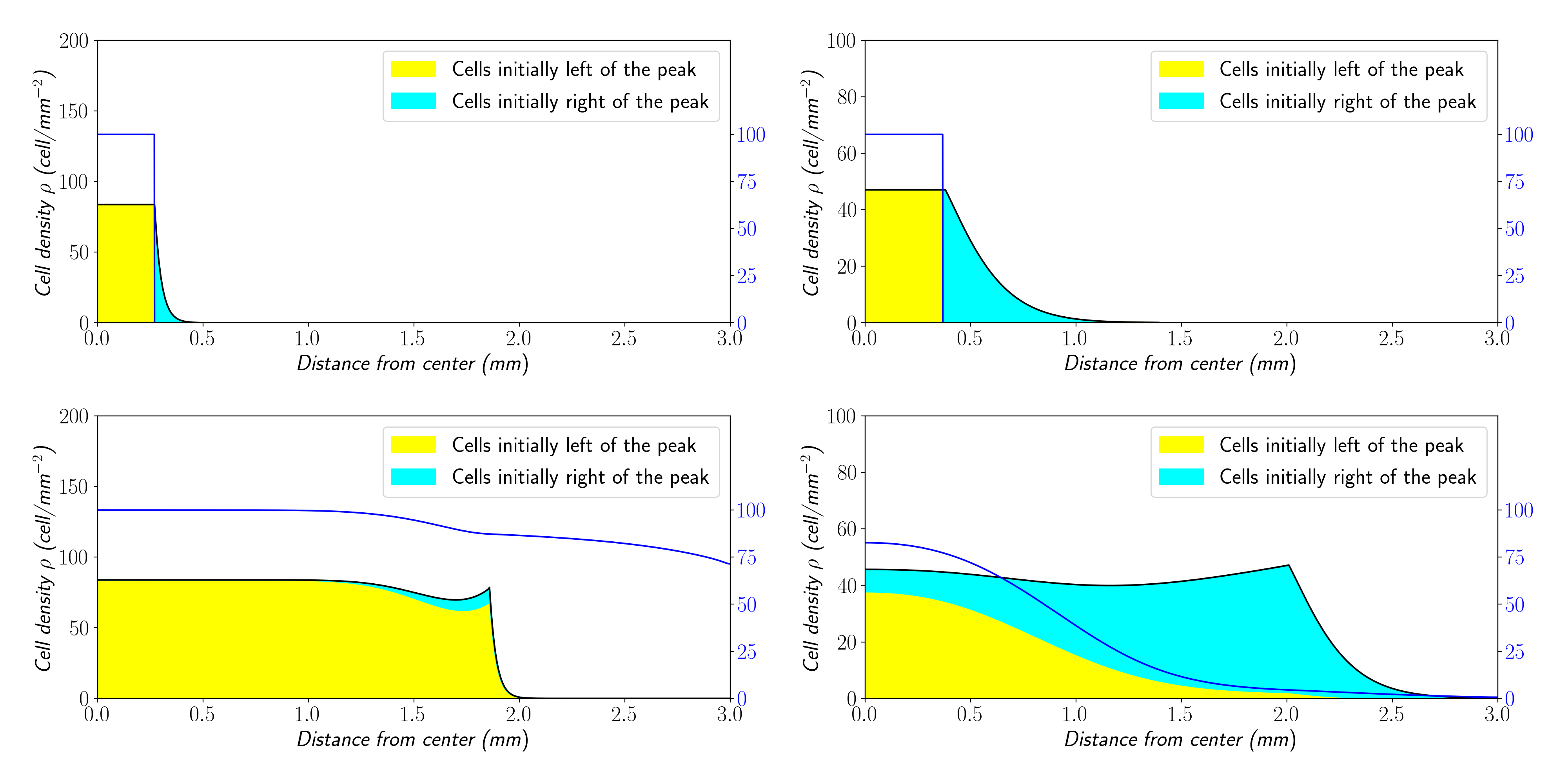

Supplement: Figure 7—source data 1. [file elife-64731-fig7-data1.zip › Fig7/Fig7.png]

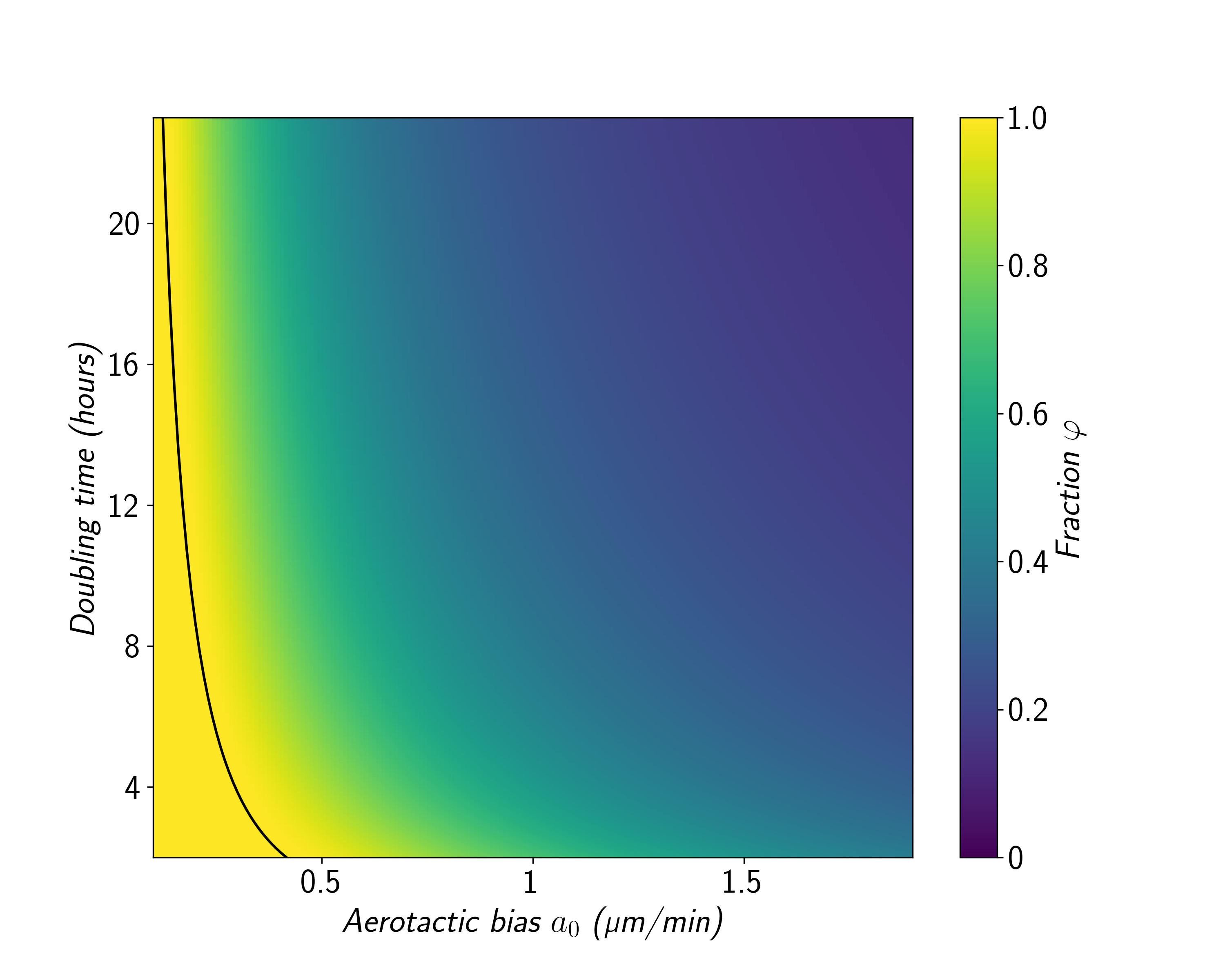

Supplement: Figure 7—figure supplement 2—source data 1. [file elife-64731-fig7-figsupp2-data1.zip › FigSI19/FigSI19.png]

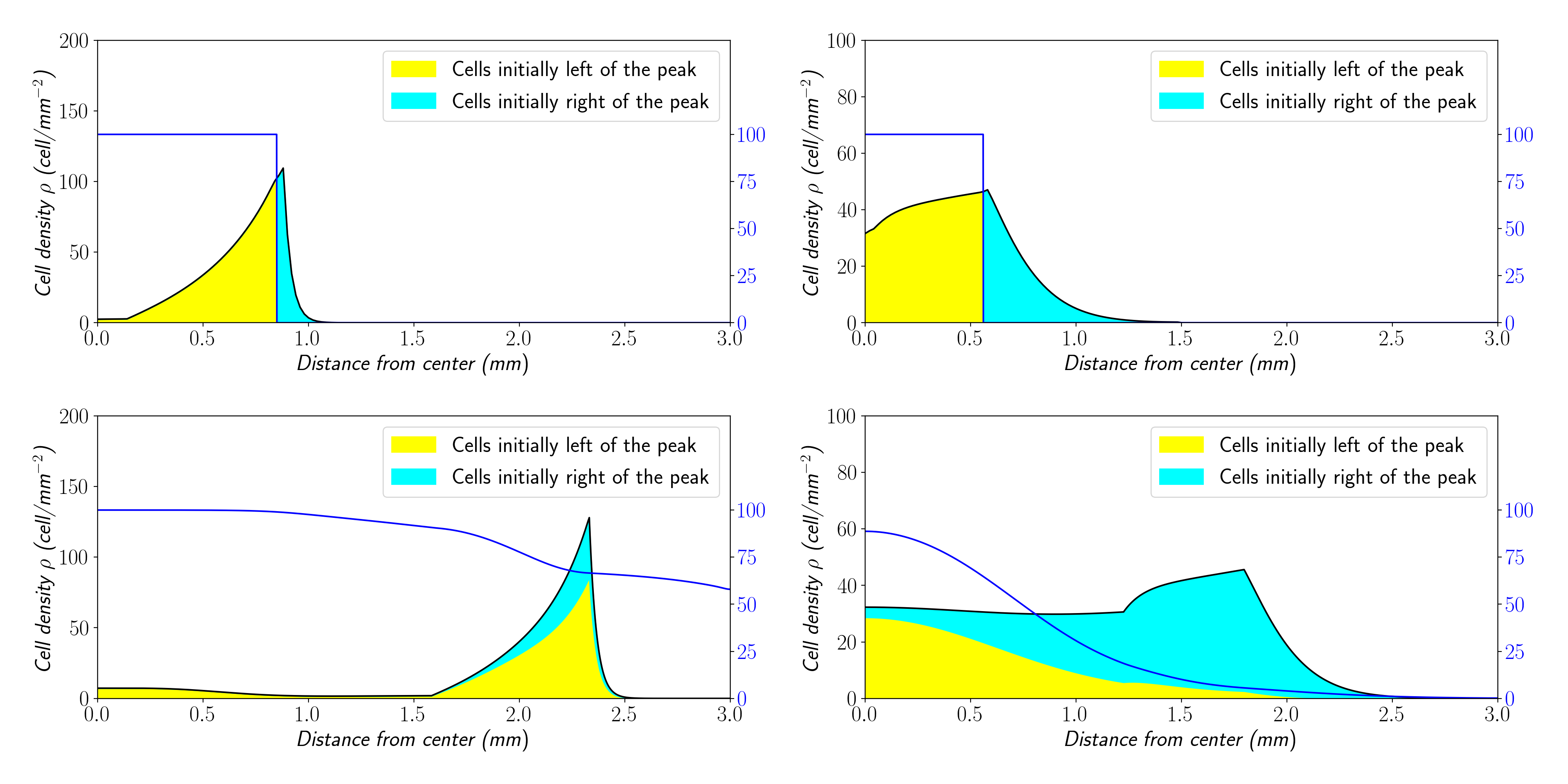

Supplement: Figure 7—figure supplement 3—source data 1. [file elife-64731-fig7-figsupp3-data1.zip › FigSI20/FigSI20.png]
